# Scoping review of climate change and mental health in Germany – Direct and indirect impacts, vulnerable groups, resilience factors

**DOI:** 10.25646/11656

**Published:** 2023-09-06

**Authors:** Nadja Gebhardt, Katharina van Bronswijk, Maxie Bunz, Tobias Müller, Pia Niessen, Christoph Nikendei

**Affiliations:** 1 Centre for Psychosocial Medicine at University Hospital Heidelberg Department for General Internal Medicine and Psychosomatics Heidelberg, Germany; 2 Psychologists/Psychotherapists for Future (registered association) Bingen, Germany; 3 Faculty of Medicine and University Hospital Cologne Institute of General Practice Cologne, Germany; 4 University of Cambridge Department of Politics and International Studies Cambridge, United Kingdom; 5 The New Institute Future of Democracy Working Group Hamburg, Germany; 6 Yale University Department of Political Science New Haven, USA; 7 Fraunhofer Institute for Systems and Innovation Research ISI Karlsruhe, Germany

**Keywords:** CLIMATE CHANGE, MENTAL HEALTH, MENTAL WELL-BEING, RESILIENCE, STRUCTURAL PREVENTION

## Abstract

**Background:**

Climate change is a major threat to human health and has direct and indirect impacts on the human psyche.

**Methods:**

To assess the state of knowledge on the impact of climate change on mental health in Germany, a scoping review was conducted for the focus topics extreme weather events, temperature increase, intra-psychological processing, sociological aspects, and resilience factors. Ten studies met the inclusion criteria of the searches in the databases Academic Search Complete, CINAHL, PubPsych, PubMed, and PsychInfo. The majority of the studies looked at correlative relationships in a cross-sectional design.

**Results:**

There are indications of an accumulation of psychiatric disorders after extreme weather events; in addition, the risk of suicide increases with higher temperatures and it appears there is an increase in aggressive behaviour. The majority of people surveyed in Germany report concerns about the consequences of climate change, although these currently rarely lead to clinically significant impairments in mental health.

**Conclusions:**

Overall, the evidence for Germany must be classified as insufficient. In addition to the absolute priority of climate protection (mitigation) by reducing emissions, there is a particular need for additional research with a focus on vulnerable groups and possibilities for prevention and adaptation.

## 1. Introduction

Ongoing climate change represents one of the main threats to humankind and affects the human psyche on several levels. While extreme weather events and the increase in average global temperature directly influence our mental well-being, our awareness of the already noticeable and future drastic consequences of climate change indirectly influences our psychological stability, our emotional experience, and our resulting behaviour. The identification of vulnerable groups as well as resilience factors at the individual and societal level is of particular importance with regard to maintaining the mental health of the whole population. Internationally, climate change-related food insecurity and migration are additional significant mental stressors [1]. As these factors do not (yet) have a noticeable impact within Germany, they are not considered in more detail in this article with its methodological focus on Germany.

### 1.1 Extreme weather events and psychological consequences

In the course of climate change, extreme weather events will become more frequent. In Germany, more frequent heavy rainfall events, heatwaves, and storms are expected [2, 3]. A detailed analysis of the psychological effects of extreme weather events in the context of somatic-general health and society can be found in the corresponding article of this status report by Butsch et al. [4]. Previous international studies have found an increase in post-traumatic symptoms, depressive and anxious symptoms, as well as heightened levels of substance use disorders in the aftermath of severe extreme weather events (e.g. heavy rainfall resulting in flooding) [5, 6]. According to international studies, whether and to what extent such extreme weather events contribute to the development and aggravation of psychiatric disorders depends on various factors. These include the type, duration, and severity of the event, the resulting physical harm, the immediate threat to one’s own life or the life of a loved one, the influence of the event on social networks, and any aid received. The subjective significance of the event and the correspondence with other biographical experiences is just as relevant as whether one’s own social existence was affected through the destruction of one’s home or personal infrastructure or the loss of income [6, 7]. In particular, persons of female sex and persons with pre-existing psychiatric disorders are considered vulnerable to the (re)occurrence and development of further psychiatric disorders as a result of an extreme weather event [8].

### 1.2 Direct effects of temperature increase on the psyche

Heat-related effects on physical health are discussed by Winklmayr et al. [9] in this status report. However, the climate change-related increase in heatwaves and days with extreme heat also has a direct impact on mental health. In international, large-scale epidemiological studies, a correlation was shown between milder temperatures, close to a comfortable 21°C, and more frequent occurrence of socially compatible character traits, such as openness and extra-version [10]. Hot days and heatwaves, on the other hand, lead to more aggressive and hostile behaviour [6, 11], which is also reflected in an increase in delinquencies, e.g. assaults, homicides, rapes, and robberies [12]. Correlations have been reported for the general population, in Germany, and internationally, between isolated rises in temperature and an increased suicide risk on the following day [13, 14]. In addition, the presence of a psychiatric disorder seems to increase vulnerability to the stressful effects of heat: in patients with dementia, bipolar disorder, or schizophrenia, a significant association between an increase in daily mean temperature and an increase in mortality can be observed [15].

### 1.3 Perception and intra-psychological processing of climate change

Awareness and realisation of the impacts of climate change can cause a variety of negative affects [16], including clinically significant psychological distress. The emotional reactions to climate information are discussed in research under terms such as climate/eco-anxiety, climate/eco-anger, solastalgia, ecological grief, ecological guilt, eco/climate depression, or climate distress [17]. However, a consistent operationalisation of the terms is still lacking, which makes it difficult to compare the results of studies [17]. The construct of climate anxiety, operationalised by the Climate Anxiety Scale, has been studied most frequently to date [18]. However, there are also questionnaires, among others, on eco-anxiety [19], climate worry [20], solastalgia [21], eco-grief, and eco-guilt [22]. The focus of scientific evaluation to date has been on links between emotional reactions to climate change and risk perception [23], climate protection behaviour [24, 25], repression/denial of climate change [26, 27], as well as protest behaviour [25, 28] and psychological distress [6, 29]. International studies agree that climate-related concern is widespread, but clinically significant symptoms are significantly less common [11, 30, 31].

### 1.4 Sociological aspects of the psychological consequences of climate change

Sociological factors play a central but often neglected role in the assessment of the psychological effects of a challenge to society as a whole, such as climate change. By sociological factors we mean diverse social contexts that influence individual and collective experience, reflection, and decision-making. While many sociological factors correlate with demographic categories such as gender, age, socio-economic status, and ethnicity, our definition also comprises organisational forms, social practices, geographic specifics, physical and ideational infrastructures, cultural norms, and political decision-making structures [32]. This is particularly important for dealing not only individually but also collectively with psychological impairments caused or exacerbated by climate change. Group determinants of mental health and illness are important in climate-related epidemiology, risk assessment, and resilience. Population subgroups that are at higher risk of experiencing negative health effects due to existing structural disadvantages and vulnerabilities are also proportionally more affected by climate change and its mental health effects [33]. International studies show that older population subgroups, for example, are more affected by psychiatric disorders as a result of extreme weather events [34]. Children, on the other hand, show a significantly increased vulnerability to the psychological consequences of extreme weather events such as floods and hurricanes [33]. Girls are a particularly vulnerable group, at increased risk of developing anxiety disorders and substance abuse after experiencing natural disasters [33].

### 1.5 Resilience factors for mental stability in the context of climate change

In addition to the risk factors and vulnerabilities that have an influence on the development of psychiatric disorders, there has been little research to date on the protective factors that relate specifically to climate change-related mental distress. According to the differentiation by Clayton [29], protective factors can be found for direct, i.e. acute events such as extreme weather events, and for indirect effects of climate change. With regard to the direct effects of climate change, the resilience factors are largely similar to those for post-traumatic stress disorder (PTSD) and include personality traits such as higher self-esteem and a more pronounced sense of coherence (the feeling that the world and oneself are understandable and predictable), individual coping strategies such as meaning-focused coping and successful emotion regulation strategies, as well as environmental factors such as family support and support in the wider social environment [35]. Resilience factors for indirect events, on the other hand, have hardly been researched so far. Initial findings indicate that personal characteristics such as gender or political orientation can, by contributing to psychological resilience, lead to relief or faster recovery from psychological distress [27].

### 1.6 Investigating the impact of climate change on mental health in Germany

While several reviews have been published internationally on the topics described, there are only a few research papers dealing with the effects of climate change on mental health in Germany. To gain an overview of the existing evidence and the additional need for knowledge, a scoping review was conducted for each of the aspects of the effects of climate change for Germany described above. In order to provide context for the small number of publications expected by the authors, international reviews and findings were included for the discussion of the identified results. The aim was to develop a comprehensive overview of the state of research in order to derive recommendations for mitigating the negative consequences of climate change on the mental health of the German population.

## 2. Methods

The procedure for preparing the scoping review followed the Preferred Reporting Items for Systematic Reviews and Meta-Analyses extension for Scoping Reviews (PRISMA-ScR) [36]. Reviews of international literature on the individual aspects were also identified using systematic searches. Relevant publications without peer review were specifically selected on the basis of the authors’ prior knowledge.

### 2.1 Scoping review

Between 05.09.2022 and 30.09.2022, the databases Academic Search Complete, CINAHL, PubPsych, PubMed, and PsychInfo were searched for scientific papers that contained one of the possible combinations of search queries in the title or abstract. No restriction was made on the date of publication. All search queries consisted of one climate change or weather-related term, such as ‘climate change’ or ‘heat wave’, the specification ‘German’ or ‘Germany’, and a search term specific to the focus topic. The latter was, for example, ‘post-traumatic stress disorder’ for extreme weather events, ‘aggression’ for temperature increases, ‘climate anxiety’ for intra-psychological processes, ‘resilience’ for resilience factors and ‘social infrastructure’ for sociological aspects. Only studies published in peer-reviewed journals were included. The exact search queries for the databases per focus topic are shown in Annex Table 1. No review protocol was published.

The database searches were carried out by the first author (NG) for all topics. The titles and summaries of the studies found were reviewed by the authors per focus topic (extreme weather events: MB, temperature increase: CN and NG, intra-psychological processing: KB, resilience factors: PN, sociological aspects: TM). Ambiguities were discussed by the whole team until a consensus was reached. Articles were excluded if they were not related to climate change; were not related to the focus topic; used a qualitative research design; were reviews, commentaries, or other articles not presenting new data. The selected studies were transferred into a standardised table by the authors. For this purpose, a table with the relevant variables was created by the research team before data extraction began. The following information was recorded: Data source; (sub-)population; number of study units; region in Germany; time period considered; type of study; phenomena/variables studied; tools; results. If studies reported data from several countries, only the data related to Germany were included in the table.

### 2.2 Review of international literature

Internationally published reviews on the focus topics of this paper were also compiled between 05.09.2022 and 30.09.2022 without a specification for Germany and using the same search strategy as the scoping review. These were included in the discussion in order to be able to meaningfully contextualise the studies reported for Germany in the results. For this purpose, the search query was appended with ‘systematic review OR meta-analysis OR meta analysis OR literature review OR scoping review’. These search queries can also be seen in Annex Table 1.

## 3. Results

**Annex Figure 1:**
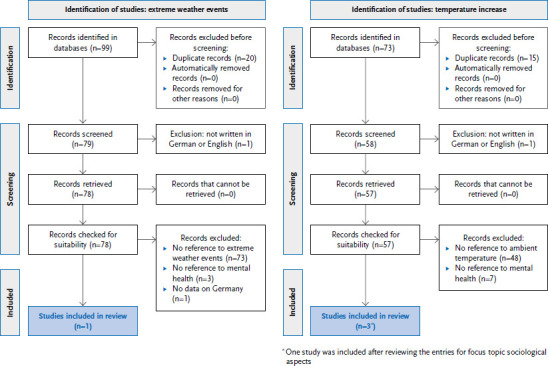

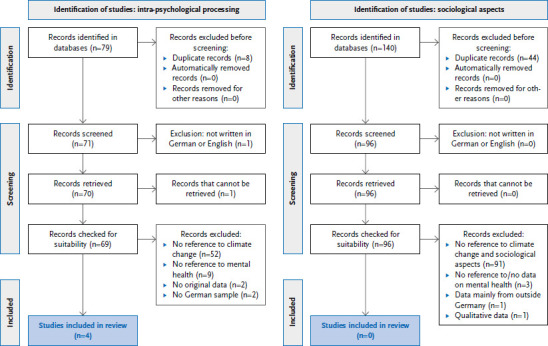

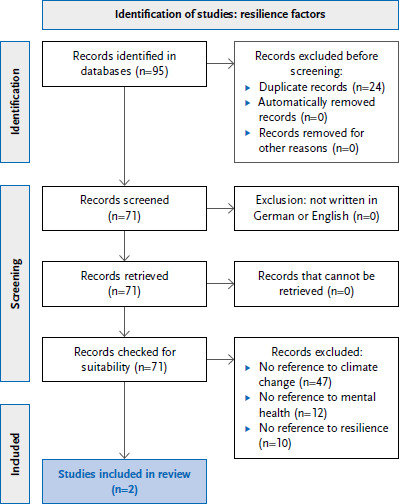
Flow charts of the literature selection per focus topic Annex Figure 1a (left) Extreme weather events Annex Figure 1b (right) Temperature increase Annex Figure 1c (left) Intra-psychological processing Annex Figure 1d (right) Sociological aspects Annex Figure 1e Resilience factors

In total, the search queries for Germany yielded 486 results across all focus topics, 111 of which were duplicates, so that 375 studies were screened for their relevance. Of these, 365 were excluded, so that ten studies were included in the final evaluation. Various study results from one article [37] were included in the results for both the focus topics intra-psychological processing and resilience factors, so that the presentation of results refers to ten studies from nine articles. Table 1 provides an overview of the results of the included studies; the PRISMA flowcharts [38] of the article selection per topic can be seen in Annex Figure 1.

### 3.1 Extreme weather events and psychological consequences in Germany

The initial search yielded 99 results, 20 of which were duplicates and one article that was not written in German or English, so that title and abstract were screened for 78 studies. Of these, 73 were excluded because they were not publications related to extreme weather events, three others were excluded because they were not related to the psychological effects of extreme weather events, and one review paper without data from Germany. Ultimately, one study was included and reviewed.

Otto et al. [39] used cross-sectional questionnaire data to examine the effects of the flood disaster in Saxony in 2002 in n=112 persons affected. Of these, 23% screened positive for the presence of PTSD, 13% for depression and 11% for anxiety disorders. Predictive for a higher burden of post-traumatic symptoms was the feeling that one’s life was in danger, that one had suffered personal losses, and that one’s own future had been destroyed. The perceived danger to one’s life as well as the assumption of a destroyed future were also predictive for stronger symptoms of anxiety and depression. A personal belief in a just world may have had a protective effect on the expression of symptoms of anxiety and depression, as pointed out by the authors of the study.

### 3.2 Direct effects of temperature increase on the psyche in Germany

The initial search yielded 73 results, 15 of which were duplicates and one article in a language other than German or English, so that title and abstract were screened for 57 studies. Of these, 48 were excluded because they were not publications related to ambient temperature and its effect on the psyche. Seven more studies were excluded because they were not related to mental health. In addition, one study from the database query for the topic ‘sociological aspects’ met the inclusion criteria and was also included in the final synthesis, so that three studies were included. All included studies looked cross-sectionally at associations between psychological variables and temperatures on the same day or in the preceding days. Müller et al. [46] reported a 0.9% increase in suicide rates for every 1°C increase in temperature in spring and summer, but not in autumn and winter. Schneider et al. [42] calculated a 5.7% increase in suicide risk for temperature jumps of 5°C the previous day in summer, autumn, and winter, but not in spring, with the risk being particularly high for older people. One study used electronic health records for patients in mental health facilities to show a significant association between daily maximum temperatures above 30°C and aggressive incidents [40].

### 3.3 Perception and intra-psychological processing of climate change in Germany

The initial search yielded 79 results, eight of which were duplicates and one article in a language other than German or English, so that title and abstract were screened for 70 studies. One article was not retrievable, 52 were excluded because they were not publications related to climate change. Nine further studies were excluded because they were not related to mental health, two studies did not collect data, and two other studies did not have a German sample. Ultimately, four studies met the inclusion criteria. In all included studies, cross-sectional correlations between psychological variables and subjective well-being were investigated. Lippold et al. [43] compared anxiety due to climate change with anxiety in the context of the COVID-19 pandemic. In the other studies, the focus was on assessing the emotional impact of climate change. Based on questionnaire data, the results agreed that hardly any clinically significant psychological distress currently arises from the emotional confrontation with climate change [37, 44, 45]. However, children and adolescents are considered vulnerable groups for the development of a clinically manifest psychiatric disorder in the event of an increase in climate change impacts [37]. Lippold et al. [43] found a below-average expression of reported anxiety due to climate change in an international comparison. Wullenkord et al. [45] report more pronounced climate change anxiety among women; furthermore, participants reported less pronounced climate anxiety when they perceived the global and personal consequences of climate change, as well as their own complicity in causing it, to be lower. Climate change-related perceived stress correlates positively with a less secure attachment style, a lower availability of regulating self-functions and a less pronounced sense of coherence, i.e. the feeling that the world and oneself are understandable and predictable [44].

### 3.4 Sociological aspects of the psychological consequences of climate change in Germany

The initial search yielded 140 results, 44 of which were duplicates, so that title and abstract were screened for 96 studies. Of these, 91 were excluded because they were not publications on sociological aspects related to climate change. In addition, two studies were excluded because they were not related to mental health, one because the data were mainly collected outside of Germany, one because the data were qualitative in nature, and one because the references to mental health were not supported by data. Thus, no study could be included as a quantitative research paper on the sociological aspects of the psychological consequences of climate change in Germany.

### 3.5 Resilience factors for mental stability in the context of climate change in Germany

The initial search yielded 95 hits, 24 of which were duplicates, so that title and abstract were screened for 71 studies. Of these, 47 were excluded because they were not publications related to climate change, twelve were excluded because they were not related to psychological impacts, and ten were excluded because they were not related to resilience or coping mechanisms. Two studies were included and reviewed.

In the two studies included, cross-sectional design identified coping strategies with regard to climate change-related psychopathologies. In a study by Wullenkord and Reese [27], self-protective strategies and their correlations in dealing with the effects of climate change were analysed with a questionnaire newly designed for this purpose. The existence of different coping strategies could be shown, such as avoidance, rationalisation, or the denial of the global and personal consequences of climate change as well as one’s own role in causing it. Furthermore, these strategies were more pronounced in men and in those with a right-wing political orientation; only avoidance was reported to be more pronounced in women than in men. Klöckner et al. [37] studied 9- to 14-year-old pupils and were able to show that emotional reactions to climate change correlated positively with more knowledge about how to mitigate it. The authors interpreted this as an indication that children who are overwhelmed with climate change withdraw emotionally and consequently absorb less information on the topic. There was no correlation between the type of climate change-related emotions and general well-being.

## 4. Discussion 4.1

### Strengths and limitations

The present review provides a comprehensive overview of the literature on the effects of climate change on mental health in Germany, drawing on a total of five systematic literature searches in five databases. This broad search strategy is contrasted by a highly insufficient number of studies, so that the generalisability of the results is limited. In order to derive meaningful recommendations, the results obtained are therefore considered alongside surveys and studies without peer review from Germany, as well as internationally published reviews on the focus topics of this article, compiled by systematic literature research. However, the state of knowledge remains insufficient after considering this set, particularly with regard to successful adaptation [48]. The search terms used were adapted for each focus topic in order to include as many relevant publications as possible, but it cannot be ruled out that additional studies might have been included if other combinations of search terms had been used. The results of qualitative studies were not included in this review due to the methodological approach followed. However, given the many connections between climate change and mental health that remain to be clarified, they provide many complementary results, such as a better understanding of the connections between climate change-related emotions and general well-being or resilience in relation to psychological distress caused by climate change [49–52].

### 4.2 Evidence and need for knowledge

When evaluating the results on the effects of climate change on mental health in Germany, it becomes apparent that there is a large discrepancy between the data and knowledge available for Germany and international findings. Both nationally and internationally, there is a great need for further scientific knowledge on the psychological effects of the climate crisis and possible adaptation strategies.

#### Common aspects across the focus topics

The effects of climate change on mental health in Germany have so far been insufficiently studied, both cross-sectionally and longitudinally. Previous international findings on the focus topics investigated for Germany in this article come largely from Australia, Canada, and the USA. Only a small proportion of the studies relate to the European population [48]. In addition, relevant constructs, such as intra-psychological processing and resilience factors, are not distinctly operationalised, and validated assessment tools are often lacking. This makes it difficult to compare the results and derive recommendations. Different methodological approaches and evaluation tools are also used to record psychological distress patterns. Often, mental health burden is recorded by means of questionnaires. However, these are often not clinically validated, i.e. no clinical diagnoses are made by clinical experts, and no validated structured clinical interviews corresponding to manuals of psychiatric disorders are conducted (e.g. the structured clinical interview according to DSM-V, the Diagnostic and Statistical Manual of Mental Disorders [53]). A special focus of future research should be on the mental health burden for vulnerable groups, of which there are hardly any findings for Germany. An increased vulnerability among children and adolescents, older persons, those with pre-existing psychiatric disorders or low socioeconomic status can be assumed in correspondence with international studies [11, 48, 54]. The same applies to people who are increasingly exposed to the consequences of climate change either directly, through extreme weather events, or indirectly, e.g. as activists or health professionals [33, 48].

#### Extreme weather events and psychological consequences

The data found in a regional sample in Germany [39] are consistent with findings in international literature on post-traumatic stress symptoms, depression, and anxiety in the aftermath of a flooding event [5, 11, 55, 56]. Floods can exacerbate pre-existing psychiatric disorders [57]. This is reflected in a higher prescription rate of psychotropic drugs such as sedatives, hypnotic drugs, or antidepressants after floods and storm surges [58, 59]. Those individuals who have to be relocated as a result of the flooding have a significantly higher risk of a subsequent psychiatric disorder, which can still be observed a year after the event [60]. Children and adolescents in particular show increased vulnerability to the effects of extreme weather events because they have fewer coping strategies and their more pronounced neuroplasticity (changes in cerebral structures and functions in response to external stimuli) makes them more susceptible to stress-induced neuroanatomical and endocrine changes [61, 62]. In international literature, Mambrey et al. [33] identified the following risk factors for mental health outcomes in children and adolescents: intra-family conflict, little social support, loss of social network due to relocation, and low socioeconomic status of parents.

#### Direct effects of temperature increase on the psyche

The increased prevalence of suicides reported for Germany at higher daily temperatures compared to the previous day has also been shown in international reviews, which found a correlation of suicide rates with an increase in daily temperatures compared to the previous day and with higher daily temperatures in general [13, 14]. The fact that this correlation is consistently reported for Germany for summer, but not for the colder seasons, could be related to the average temperature in autumn, spring, and winter being close to or below 21°C (for now), a daily average temperature considered comfortable. However, the increase in daily average temperatures will continue as climate change progresses [63]. The question remains whether suicide rates can be reduced through education and heat protection. Increased aggressive behaviour, as shown in psychiatric institutions by Eisele et al. [40], has been shown internationally for the general population [6, 11]. In addition to the effects on suicide risk, an increase in admissions to psychiatric hospitals at higher daily average temperatures has been reported in international studies [48]. A study conducted in the USA found fewer positive and more negative emotions in subjects when daily average temperatures exceeded 21°C [11]. People with pre-existing psychiatric disorders, children, and adolescents are particularly vulnerable to the effects of heat on mental health [48].

#### Perception and intra-psychological processing of climate change

As the negative impacts of climate change on mental health will increase in the future, there is a need for a better understanding of the transition from an adequate emotional response to climate change to clinically relevant mental health impairments. Surveys conducted in Germany show a high prevalence (40–73%) of general anxiety, sadness, and anger in all age groups, in line with international study results [64–67]. These feelings increased significantly nationwide after the July 2021 floods (by 20 percentage points [66]). It can be assumed that the media coverage of the floods had an influence on intra-psychological processing and the negative affects subsequently reported by the respondents. The role of the media in psychological adaptation processes to climate change and mitigation should therefore be discussed. To this end, media guidelines can serve as a recommendation for a style of reporting that neither aims to trivialise weather phenomena that occur nor to reinforce a sense of powerlessness [68]. In a report by the German Environment Agency on the emotional state of young people in the context of climate change, 26% of the respondents stated that concerns about the environment limited their sense of joy and caused sleep problems [69]. International studies show that the majority of young people, regardless of gender, consider climate impacts when making reproductive choices [47]. A better handling of climate feelings and a strengthening of resilience factors requires good psychoeducation as well as opportunities for exchange with like-minded people and the experience of collective self-efficacy through opportunities for action to achieve societal transformation and climate protection [70].

#### Sociological aspects of the psychological consequences of climate change

The current state of studies on sociological aspects of climate change-related impairments of mental health does not allow for clear statements on risk factors, effects, or possible countermeasures. In addition, there are currently no studies from the German-speaking region that examine the connection between climate change and specific sociodemographic or sociological factors and intersectional discrimination (i.e. the reinforcing effects of interdependent systems of discrimination such as patriarchy, capitalism, colonialism, ableism – the discrimination against people with limited mental or physical abilities [71–73]) in terms of mental health. In this context, sociodemographic factors such as ethnicity, family history of migration, and socioeconomic status would be of interest, as well as sociological factors such as spatial marginalisation (e.g. due to the stigmatisation and infrastructural deficiency of certain neighbourhoods in which certain ethnic, cultural, or religious groups are more strongly represented). Studies on the psychological impact of an extreme event, such as the COVID-19 pandemic, have shown that sociological factors, such as living in a neighbourhood disadvantaged in terms of health infrastructure, having few social contacts, and being part of a minority subjected to structural racism, can lead not only to higher mortality, but also to a significantly higher risk of psychological distress [74]. Climate change is expected to lead to an increase in social states of emergency due to heatwaves, supply shortages, floods, power cuts, or the collapse of public and private services. Therefore, an analysis of the different living environments in which sociological factors can have both positive and negative influences is indispensable for a psychologically sensitive approach to crisis situations caused by climate change.

#### Resilience factors for mental stability in the context of climate change

Climate change, through stressors such as heat, poor air quality, the possible loss of emotionally significant places and landscapes, and potentially even forced migration, reduces the possibilities of building psychological resilience, which underlines the importance of strengthening the existing psychological resources at the individual and collective level [11]. In the studies considered for Germany, different definitions of resilience emerged: on the one hand, factors such as biological sex are identified, which reduce the risk of developing psychological stress and thus establish resilience. On the other hand, factors that are actively protective, such as social support, are examined. Thus, when there is an increased risk, protective factors can lead to resilience. At the same time, if the risk of psychological distress is low, these protective factors are not relevant for psychological recovery after a distressing event. The classification as a resilience factor thus depends on the existing vulnerability of an individual. The results of the available studies are only of limited significance in this context. While Wullenkord and Reese [27] examine self-protective strategies as mechanisms for coping with climate change-related psychological distress, they do not consider them to be resilience factors in the sense of successful coping, rather as dysfunctional mechanisms (denial and avoidance). The factors identified by Klöckner et al. [37] similarly are considered reactive mechanisms and not successful coping. In contrast, a report by the German Environment Agency, through qualitative interviews with young climate activists, identifies the following resilience factors: knowledge on how to deal with psychological distress, positive cognitive assumptions, support and appreciation, as well as social and societal support structures [69]. The international literature on resilience factors mainly refers to individual extreme weather events [35]. In contrast, the indirect factors, such as the effects of climate change on the psyche in regions that are currently not (yet) acutely affected by the consequences of climate change, have not yet been studied. Chen et al. [75] were able to identify an extensive list of resilience factors. Intact family structures and a higher level of education in particular have an active protective effect against psychological stress after extreme weather events. This indicates that it is not so much the individual’s psychological coping abilities that enable successful handling of psychological distress, but rather the societal and social embedding of the individual. It is therefore necessary to promote resilience factors on a collective or political level.

### 4.3 Recommendations

The recommendations listed in Table 2 result from the collected findings presented in this article. As there is still a great need for research in Germany, the identified fields of action result from a comparison with international literature. The measures derived were adapted to the German context according to the authors’ assessment. It is assumed that findings valid for the Anglo-American region can largely be transferred to the European context. Further reference can therefore be made to corresponding publications, for example those by the American Psychological Association [11]. The position paper on climate change and mental health of the German Association for Psychiatry, Psychotherapy and Psychosomatics (Deutsche Gesellschaft für Psychiatrie und Psychotherapie, Psychosomatik und Nervenheilkunde, DGPPN) [76] was also used as a source of information in the preparation of the recommendations and its considerations were adapted to a public health context.

The recommendations made here refer specifically to health promotion through medical-psychotherapeutic treatment, structural, and behavioural prevention, thus focusing on the adaptation to the impact of climate change on mental health. An expansion of the psychiatric and psychotherapeutic care infrastructure appears all the more urgent as the current situation already falls far short of demand [77]. The inclusion of climate change and its impact on mental health in the training of psychotherapeutic professions is also aimed at the practitioners themselves: they must first find their own way to intra-psychologically process knowledge about climate change and its effects; only then can they ensure competent treatment of patients with climate change-related issues [78–80].

Considering the numerous negative consequences of climate change for the human psyche, it must be emphasised once again in all clarity that, in addition to the development of adaptation strategies, climate protection measures are imperative and have top priority in order to minimise an increase in psychological risks. In this sense, climate protection is the most effective form of health protection [81]. The healthcare sector, which in Germany is responsible for 5.2%–6.7% of national greenhouse gas emissions depending on estimates [82, 83], should be particularly committed to the health of the population and thus also to climate protection.

### 4.4 Conclusion

The effects of climate change on mental health are diverse and depend on individual and societal factors. Extreme weather events and rising average temperatures have a direct influence on mental health, an indirect influence is exerted by awareness of the human contribution to climate change and its consequences. For Germany, the state of research on these processes is highly insufficient, and both in Germany and internationally, there is a particular lack of knowledge on how to achieve successful adaptation to the effects of climate change on mental health. This should be a focus of further research. In addition to adaptation, mitigation must also be seen as a societal task. The healthcare system has a special role to play here, since it accounts for a considerable share of human greenhouse gas emissions. A successful reduction of this share would simultaneously protect the health of patients.

## Key statements

- Extreme weather events, rising temperatures, and climate change awareness seem to have a negative impact on the mental health of the population in Germany.
- Heat and sharp temperature rises lead to increase in suicide rates and aggressive behaviours.
- Experiencing extreme weather events increases the risk of developing psychiatric disorders (post-traumatic stress disorder, anxiety disorders, and depression).
- Intra-psychological processing of climate change leads to many worries, but seldom to clinically relevant mental health impairments.
- Psychological resilience factors can play a crucial role in adapting to the impacts of climate change.
- In order to promote psychological resilience and adequately counter the psychological distress resulting from climate change, there is urgent need to expand our knowledge.
- For all topics examined, the state of knowledge for Germany is to be regarded as insufficient, which makes it difficult to draw final conclusions.
- Sociological aspects are decisive factors for adequate healthcare in case of extreme events.
- In addition to reducing our emissions, we need to expand social education and mental healthcare for vulnerable groups, as well as for those affected by extreme weather events.

## Figures and Tables

**Table 1:** Results of the Scoping Review on the impact of climate change on mental health in Germany

Focus topic: Extreme weather events and psychological consequences in Germany								
Authors, publication year	Data source	(Sub-) population	Number (n)	Region, period under consideration	Type of study	Phenomena/variables studied	Tools	Results
Otto et al. [39], 2006	Questionnaire survey	Victims of the 2002 flood disaster in Saxony	112	Saxony, 2002–2003	Cross-sectional design	PTSD, depression, anxiety disorders, general psychological distress	Validated, psychological questionnaires (IES-R, BDI, BAI, BSI)	Symptoms of PTSD: n=26, symptoms of depression: n=15, marked anxiety: n=12. Those who reported that their lives had been in danger, that they had suffered personal losses and that they expected their future to be affected had significantly more symptoms of PTSD. The likelihood of depression and anxiety also increased when life was in danger and the future was expected to be affected.

Focus topic: Direct effects of temperature increase on the psyche in Germany								
Authors, publication year	Data source	(Sub-) population	Number (n)	Region, period under consideration	Type of study	Phenomena/variables studied	Tools	Results
Eisele et al. [40], 2021	Electronic health data	Patients in psychiatric institutions	164,435	Baden-Württemberg, 2007–2019	Correlative relationships in a cross-sectional design	Aggressive behaviour	SOAS-R	At daily maximum temperatures >30°C significantly more aggressive incidents, number increases linearly with temperature; no significant correlation of daily maximum temperature with number of coercive measures
Müller et al. [41], 2011	Official data	(Attempted) suicides in the general population	2,987	Middle Franconia, Bavaria, 1998–2005	Correlative relationships in a cross-sectional design	Attempted suicide, completed suicide	Police protocols	Significant increase in the number of suicide attempts and suicides with rising temperatures and increased sunlight; no significant association with humidity, gender, motive or method of suicide (attempt)
Schneider et al. [42], 2020	Official data	Suicides in the general population	10,595	Bavaria, 1990–2006	Correlative relationships in a cross-sectional design	Suicide	Not applicable	Significant increase in the number of suicides by 5.7% for temperature rises >5°C the previous day in summer, autumn, and winter, not in spring; 9.0% for persons >65 years of age

Focus topic: Perception and intra-psychological processing of climate change in Germany								
Authors, publication year	Data source	(Sub-) population	Number (n)	Region, period under consideration	Type of study	Phenomena/variables studied	Tools	Results
Klöckner et al. [37], 2010^1^	Questionnaire survey	9- to 14-year-old pupils (representative), years 4–7 at mainstream schools (no special schools)	2,013	Hesse, 2010	Correlative relationships in a cross-sectional design, multithematic panel study	Emotional reactions, environmental behaviour, general well-being	Self-generated questionnaire with 1 item each on feelings, well-being, knowledge of action, possibilities for action	Most children report ethically motivated, self-referential emotions, e.g. a guilty conscience about climate change. Girls mentioned consequence-based emotions (fear, grief) more often than boys, but coping-centred non-emotional expressions (e.g. disinterest) less often. With regard to climate change, the proportion of coping-centred expressions, as well as other non-emotional expressions, increased with children’s age. Ethically motivated, self-referential emotions became less frequent with increasing age. Well-being is hardly affected by climate change-related emotions.
Lippold et al. [43], 2020	Online survey	General population	3,469	Germany, 03/2020	Multivariate linear regression models	Fear of coronavirus, refugees, and climate change	rRST-Q, BFI, and other custom-made items	Compared to international respondents, those in Germany report less fear of climate change than respondents from other countries. Fear of climate change correlates negatively with a conservative political attitude.
Schwaab et al. [44], 2022	Questionnaire survey	Medical students	203	Heidelberg, 05–12/2021	Correlative relationships in a cross-sectional design	Psychological stress in general and due to climate change, resilience factors	Climate change questions from the European Social Survey, PHQ-9, GAD-7, PTSS-10, PSQ-20, RQ, OPD-SF, SOC-13	60% of participants report being (very) concerned about climate change, but clinical symptoms (trauma, depression, anxiety) when thinking about climate change are hardly reported, although 23% report increased stress levels (PSQ-20). These correlate with a less secure attachment style, less structural integration, and a less pronounced sense of coherence.
Wullenkord et al. [45], 2021	Online questionnaire survey	General population (stratified sampling)	1,011	Germany, not specified	Correlative relationships in a cross-sectional design	Psychological stress in general and due to climate change, environmental behaviour, political orientation	Climate Anxiety Scale (German translation), PHQ-4, scales on political attitudes and attitudes towards the environment	High levels of climate anxiety are associated with high levels of anxiety and depression, avoidance of the issue in everyday life, and more awareness of the impacts of climate change and one’s own part in its genesis. Women report more climate anxiety than men, with no difference for education and income. Environmentally friendly behaviour is more pronounced for high climate anxiety scores.

Focus topic: Resilience factors for mental stability in the context of climate change in Germany								
Authors, publication year	Data source	(Sub-) population	Number (n)	Region, period under consideration	Type of study	Phenomena/variables studied	Tools	Results
Klöckner et al. [37], 2010^1^	Questionnaire survey	9- to 14-year-old pupils (representative), years 4–7 at mainstream schools (no special schools)	2,013	Hesse, 2007	Correlative relationships in a cross-sectional design	Reporting of climate change, emotional concern, behavioural change	Custom-made questionnaire	The type of climate change-related emotions is not related to overall well-being. However, children who have emotions such as sadness about climate change and at the same time have ideas on how to mitigate it (e.g. taking the train instead of driving) report lower well-being. Children who report emotional reactions to climate change have more actionable knowledge about climate protection than children who emotionally withdraw from the climate discussion.
Wullenkord and Reese [27], 2021	Online questionnaire survey	General population (convenience sample)	Study 1: n=354 Study 2: n=453	Germany, not specified	Correlative relationships in a cross-sectional design	Self-protective strategies, PEB, sociodemographic background (age, gender, education, income), political orientation	Climate Self-Protection Scale (CSPS)	Different self-protective strategies exist in coping with the emotions and fears triggered by climate change. A male gender and a right-wing political orientation are related to coping strategies such as avoidance, rationalisation, or denial of the global and personal consequences of climate change, as well as one’s own complicity in causing it.

PTSD=post-traumatic stress disorder, IES-R=Impact of Event Scale Revised, BDI=Beck Depression Inventory, BAI=Beck Anxiety Inventory, BSI=Brief Symptom Inventory

SOAS-R=Staff Observation Aggression Scale Revised

^1^ For each focus topic, the partial results of interest from the study by Klöckner et al. [37] are reported.

rRST-Q=revised Reinforcement Sensitivity Theory Questionnaire, BFI=Big Five Inventory, PHQ=Patient Health Questionnaire, GAD=Generalised Anxiety Disorder Scale, PTSS=Posttraumatic Stress Scale, PSQ=Perceived Stress Questionnaire, RQ=Relationship Questionnaire , OPD-SF=Operationalised Psychodynamic Diagnostics Short Form, SOC=Sense of Coherence

PEB=pro-environmental behaviour

**Table 2:** Recommendations for health promotion through medical-psychotherapeutic treatment as well as through structural and behavioural prevention in order to adapt to the negative effects of climate change on mental health in Germany

Health promotion by incorporating the impact of climate change on mental health into adaptation processes			
Measures/target group	Approach/objective	Actors	Requirements
Active participation of healthcare experts in political transformation processes	Inclusion of mental health in the identification of needs, resilience resources, and social adaptation to climate impacts in the context of political decision-making and transformation processes	Federal, state, and municipal levels	Financial and human resources at intervention level
Training those responsible for mental health issues and involving them in developing strategies for adapting and mitigating the consequences of climate change on mental health in public institutions, especially in the health sector	Protecting the mental health of the population through sustainable mitigation of the mental health impacts of climate change and adaptation	State and municipal levels	Financial and human resources, creation of positions for change agents

Health promotion through psychosocial emergency care and psychotherapeutic treatment			
Measures/target group	Approach/objective	Actors	Requirements
Training and expansion of psychosocial emergency care	Secondary prevention of long-term psychological consequences after extreme weather events	Federal and state levels	Expertise-based needs assessments, financial and human resources, establishment and expansion of existing structures
Adjustment when planning the demand for psychotherapeutic care by counselling centres and psychotherapists to the (increasing) climate change-related needs including peak demand after extreme weather events	Improving mental health across society through needs-based care and secondary prevention	Federal and state levels (politics and administration)	Expertise-based needs assessment, financial and human resources

Health promotion by means of structural prevention			
Measures/target group	Approach/objective	Actors	Requirements
Integration of knowledge about the connection between planetary and mental health into the education and training of health personnel and crisis intervention services.	Improving overall mental health in society through psychoeducation and structural prevention	Federal and state levels, those responsible for the preparation of study regulations, education and training guidelines	Teacher training, financial and human resources
Promote further research and development of interventions on mental health and the climate crisis	Improving prevention and treatment of climate change- related mental distress and disorders	Federal level, universities	Financial and human resources
Preparation of heat-health action plans, urban development changes towards sponge cities with more green spaces	Protection of vulnerable groups, promotion of mental well-being	Federal, state, and municipal levels	Financial and human resources at municipal level
Establishment of climate councils in which representatives of socially marginalised and particularly vulnerable groups advise executive and legislative bodies on local/regional measures and participate in decision-making.	Participation of social groups to identify needs, resilience resources and social adaptation to climate impacts	Federal, state, and municipal levels	Financial and human resources at the intervention level, legislative changes may be needed to grant decision-making powers

Health promotion through behavioural prevention			
Measures/target group	Approach/objective	Actors	Requirements
Public relations work on the impact of climate change on mental health, on prevention and treatment options, and on ways to strengthen individual and collective resilience	Empowerment of (potentially) affected people and the general population by informing them about individual possibilities for action and about the measures taken to protect the population.	Federal, state, and municipal levels, service providers	Financial and human resources, further research with focus on Germany, reference to this research by public relations activities

## Appendix

**Annex Table 1:** Search queries of the literature search per focus topic, formatted for PubMed. Each second query per focus topic is aimed at internationally published reviews without reference to Germany.

Focus topic: Extreme weather events and psychological consequences in Germany
((climate change[Title/Abstract]) OR (global warming[Title/Abstract]) OR (climate[Title/Abstract]) OR (weather[Title/Abstract]) OR (flood*[Title/Abstract]) OR (heat wave[Title/Abstract]) OR (extreme weather[Title/Abstract]) OR (hurricane*[Title/Abstract]) OR (tornado*[Title/Abstract]) OR (greenhouse effect[Title/Abstract])) AND ((depression [Title/Abstract]) OR (anxiety [Title/Abstract]) OR (trauma [Title/Abstract]) OR (post-traumatic stress disorder [Title/Abstract]) OR (mood disorder [Title/Abstract]) OR (suicide [Title/Abstract]) OR (substance abuse [Title/Abstract]) OR (alcohol [Title/Abstract]) OR (mania [Title/Abstract]) OR (schizophrenia [Title/Abstract]) OR (bipolar [Title/Abstract]) OR (ptsd [Title/Abstract])) AND ((german[Title/Abstract]) OR (germany[Title/Abstract]) OR (deutsch[Title/Abstract]) OR (Deutschland[Title/Abstract]))
((climate change[Title/Abstract]) OR (global warming[Title/Abstract]) OR (climate[Title/Abstract]) OR (weather[Title/Abstract]) OR (flood*[Title/Abstract]) OR (heat wave[Title/Abstract]) OR (extreme weather[Title/Abstract]) OR (hurricane*[Title/Abstract]) OR (tornado*[Title/Abstract]) OR (greenhouse effect[Title/Abstract])) AND ((depression [Title/Abstract]) OR (anxiety [Title/Abstract]) OR (trauma [Title/Abstract]) OR (post-traumatic stress disorder [Title/Abstract]) OR (mood disorder [Title/Abstract]) OR (suicide [Title/Abstract]) OR (substance abuse [Title/Abstract]) OR (alcohol [Title/Abstract]) OR (mania [Title/Abstract]) OR (schizophrenia [Title/Abstract]) OR (bipolar [Title/Abstract]) OR (ptsd [Title/Abstract])) AND ((systematic review [Title/Abstract]) OR (meta-analysis [Title/Abstract]) OR (meta analysis [Title/Abstract]) OR (literature review [Title/Abstract]) OR (scoping review [Title/Abstract]))

Focus topic: Direct effects of temperature increase on the psyche in Germany
((heat[Title/Abstract]) OR (hot weather[Title/Abstract]) OR (temperature rise[Title/Abstract]) OR (temperature regulation[Title/Abstract])OR (heat wave[Title/Abstract]) OR (heat waves[Title/Abstract])OR (rising temperature[Title/Abstract]) OR (rising temperatures[Title/Abstract])) AND ((aggression[Title/Abstract]) OR (cognition[Title/Abstract]) OR (cognitive dysfunction[Title/Abstract]) OR (crime[Title/Abstract]) OR (depression[Title/Abstract]) OR (anxiety[Title/Abstract]) OR (trauma[Title/Abstract]) OR (post-traumatic stress disorder[Title/Abstract]) OR (mood disorder[Title/Abstract]) OR (suicide[Title/Abstract]) OR (substance abuse[Title/Abstract]) OR (alcohol[Title/Abstract]) OR (mania[Title/Abstract]) OR (schizophrenia[Title/Abstract]) OR (bipolar[Title/Abstract]) OR (ptsd[Title/Abstract])) AND ((german[Title/Abstract]) OR (germany[Title/Abstract]) OR (deutsch[Title/Abstract]) OR (Deutschland[Title/Abstract]))
((heat[Title/Abstract]) OR (hot weather[Title/Abstract]) OR (temperature rise[Title/Abstract]) OR (temperature regulation[Title/Abstract])OR (heat wave[Title/Abstract])OR (heat waves[Title/Abstract])OR (rising temperature[Title/Abstract])OR (rising temperatures[Title/Abstract])) AND ((aggression[Title/Abstract]) OR (cognition[Title/Abstract]) OR (cognitive dysfunction[Title/Abstract]) OR (crime[Title/Abstract]) OR (depression[Title/Abstract]) OR (anxiety[Title/Abstract]) OR (trauma[Title/Abstract]) OR (post-traumatic stress disorder[Title/Abstract]) OR (mood disorder[Title/Abstract]) OR (suicide[Title/Abstract]) OR (substance abuse[Title/Abstract]) OR (alcohol[Title/Abstract]) OR (mania[Title/Abstract]) OR (schizophrenia[Title/Abstract]) OR (bipolar[Title/Abstract]) OR (ptsd[Title/Abstract])) AND ((systematic review[Title/Abstract]) OR (meta-analysis[Title/Abstract]) OR (meta analysis[Title/Abstract]) OR (literature review[Title/Abstract]) OR (scoping review[Title/Abstract]))

Focus topic: Perception and intra-psychological processing of climate change in Germany
((climate change[Title/Abstract]) OR (global warming[Title/Abstract]) OR (climate crisis[Title/Abstract]) OR (climate[Title/Abstract]) OR (greenhouse effect[Title/Abstract])) AND ((climate anxiety[Title/Abstract]) OR (eco anxiety[Title/Abstract]) OR (eco-anxiety[Title/Abstract]) OR (solastalgia[Title/Abstract]) OR (climate grief[Title/Abstract]) OR (eco grief[Title/Abstract]) OR (ecological grief[Title/Abstract]) OR (eco depression[Title/Abstract]) OR (climate anger[Title/Abstract]) OR (eco anger[Title/Abstract]) OR (eco-anger[Title/Abstract]) OR (eco guilt[Title/Abstract])OR(climate distress[Title/Abstract]) OR (activist burnout[Title/Abstract])OR(active hope[Title/Abstract])OR (beyond hope[Title/Abstract])OR (emotions[Title/Abstract])OR(eco-guilt[Title/Abstract])) AND ((german[Title/Abstract]) OR (germany[Title/Abstract]) OR (deutsch[Title/Abstract]) OR (Deutschland[Title/Abstract]))
((climate change[Title/Abstract]) OR (global warming[Title/Abstract]) OR (climate crisis[Title/Abstract]) OR (climate[Title/Abstract]) OR (greenhouse effect[Title/Abstract])) AND ((climate anxiety[Title/Abstract]) OR (eco anxiety[Title/Abstract]) OR (eco-anxiety[Title/Abstract]) OR (solastalgia[Title/Abstract]) OR (climate grief[Title/Abstract]) OR (eco grief[Title/Abstract]) OR (ecological grief[Title/Abstract]) OR (eco depression[Title/Abstract]) OR (climate anger[Title/Abstract]) OR (eco anger[Title/Abstract]) OR (eco-anger[Title/Abstract]) OR (eco guilt[Title/Abstract])OR(climate distress[Title/Abstract]) OR (activist burnout[Title/Abstract])OR(active hope[Title/Abstract])OR (beyond hope[Title/Abstract])OR (emotions[Title/Abstract])OR(eco-guilt[Title/Abstract])) AND ((systematic review[Title/Abstract]) OR (meta-analysis[Title/Abstract]) OR (meta analysis[Title/Abstract]) OR (literature review[Title/Abstract]) OR (scoping review[Title/Abstract]))

Focus topic: Sociological aspects of the psychological consequences of climate change in Germany
((climate[Title/Abstract]) OR (climate change[Title/Abstract])OR (climate crisis[Title/Abstract]) OR (temperature[Title/Abstract]) OR (global warming[Title/Abstract]) OR (heat build-up[Title/Abstract]) OR (heat[Title/Abstract])OR (temperature fluctuation[Title/Abstract]) OR (variations in temperature[Title/Abstract]) OR (extreme weather events[Title/Abstract]) OR (drought[Title/Abstract]) OR (flood[Title/Abstract])OR (floods[Title/Abstract])OR (flooding[Title/Abstract])OR (sea-level rise[Title/Abstract]) OR (rise in sea level[Title/Abstract])OR (hot house scenario[Title/Abstract])OR (hot bulp[Title/Abstract]) OR (ipcc[Title/Abstract])OR (political ecology[Title/Abstract]) OR (climate-related[Title/Abstract]) OR (climate justice[Title/Abstract])) AND ((mental health[Title/Abstract]) OR (mental illness[Title/Abstract]) OR (mental illnesses[Title/Abstract]) OR (mental disorder[Title/Abstract]) OR (depression[Title/Abstract]) OR (anxiety[Title/Abstract]) OR (trauma[Title/Abstract])OR (post-traumatic stress disorder[Title/Abstract]) OR (mood disorder[Title/Abstract]) OR (suicide[Title/Abstract]) OR (substance abuse[Title/Abstract]) OR (alcohol[Title/Abstract]) OR (mania[Title/Abstract]) OR (schizophrenia[Title/Abstract]) OR (bipolar[Title/Abstract]) OR (ptsd[Title/Abstract]) OR (sucidal[Title/Abstract]) OR (well being[Title/Abstract]) OR (well-being[Title/Abstract]) OR (quality of life[Title/Abstract])) AND ((emotional identification[Title/Abstract]) OR (terror management theory[Title/Abstract]) OR (communal well-being[Title/Abstract]) OR (communal well being[Title/Abstract]) OR (health infrastructure[Title/Abstract]) OR (social infrastructure[Title/Abstract]) OR (family cohesion[Title/Abstract]) OR (social determinants of health[Title/Abstract]) OR (aggression[Title/Abstract]) OR (violence[Title/Abstract]) OR (femicide[Title/Abstract]) OR (communal health[Title/Abstract]) OR (stress proliferation[Title/Abstract]) OR (disability[Title/Abstract]) OR (disabilities[Title/Abstract]) OR (lgbtq[Title/Abstract]) OR (indigene[Title/Abstract]) OR (seniors[Title/Abstract]) OR (children[Title/Abstract]) OR (youth[Title/Abstract]) OR (neurodiversity[Title/Abstract]) OR (refugees[Title/Abstract]) OR (asylum[Title/Abstract]) OR (spatial disparity[Title/Abstract]) OR (spatial disparities[Title/Abstract]) OR (neighborhood[Title/Abstract]) OR (neighborhoods[Title/Abstract]) OR (neighbourhood[Title/Abstract]) OR (neighbourhoods[Title/Abstract]) OR (racism[Title/Abstract]) OR (ethnic minority[Title/Abstract]) OR (ethnic minorities[Title/Abstract]) OR (sinti[Title/Abstract]) OR (roma[Title/Abstract]) OR (religion[Title/Abstract]) OR (islamophobia[Title/Abstract]) OR (antisemitism[Title/Abstract]) OR (antiziganims[Title/Abstract]) OR (sexism[Title/Abstract]) OR (capitalism[Title/Abstract]) OR (patriarchy[Title/Abstract]) OR (colonialism[Title/Abstract]) OR (group based discrimination[Title/Abstract]) OR (homelessness[Title/Abstract])OR (education[Title/Abstract]) OR (inequality[Title/Abstract])) AND ((german[Title/Abstract]) OR (germany[Title/Abstract]) OR (deutsch[Title/Abstract]) OR (Deutschland[Title/Abstract]))
((climate[Title/Abstract]) OR (climate change[Title/Abstract])OR (climate crisis[Title/Abstract]) OR (temperature[Title/Abstract]) OR (global warming[Title/Abstract]) OR (heat build-up[Title/Abstract]) OR (heat[Title/Abstract])OR (temperature fluctuation[Title/Abstract]) OR (variations in temperature[Title/Abstract]) OR (extreme weather events[Title/Abstract]) OR (drought[Title/Abstract]) OR (flood[Title/Abstract])OR (floods[Title/Abstract])OR (flooding[Title/Abstract])OR (sea-level rise[Title/Abstract]) OR (rise in sea level[Title/Abstract])OR (hot house scenario[Title/Abstract])OR (hot bulp[Title/Abstract]) OR (ipcc[Title/Abstract])OR (political ecology[Title/Abstract]) OR (climate-related[Title/Abstract]) OR (climate justice[Title/Abstract])) AND ((mental health[Title/Abstract]) OR (mental illness[Title/Abstract]) OR (mental illnesses[Title/Abstract]) OR (mental disorder[Title/Abstract]) OR (depression[Title/Abstract]) OR (anxiety[Title/Abstract]) OR (trauma[Title/Abstract])OR (post-traumatic stress disorder[Title/Abstract]) OR (mood disorder[Title/Abstract]) OR (suicide[Title/Abstract]) OR (substance abuse[Title/Abstract]) OR (alcohol[Title/Abstract]) OR (mania[Title/Abstract]) OR (schizophrenia[Title/Abstract]) OR (bipolar[Title/Abstract]) OR (ptsd[Title/Abstract]) OR (sucidal[Title/Abstract]) OR (well being[Title/Abstract]) OR (well-being[Title/Abstract]) OR (quality of life[Title/Abstract])) AND ((emotional identification[Title/Abstract]) OR (terror management theory[Title/Abstract]) OR (communal well-being[Title/Abstract]) OR (communal well being[Title/Abstract]) OR (health infrastructure[Title/Abstract]) OR (social infrastructure[Title/Abstract]) OR (family cohesion[Title/Abstract]) OR (social determinants of health[Title/Abstract]) OR (aggression[Title/Abstract]) OR (violence[Title/Abstract]) OR (femicide[Title/Abstract]) OR (communal health[Title/Abstract]) OR (stress proliferation[Title/Abstract]) OR (disability[Title/Abstract]) OR (disabilities[Title/Abstract]) OR (lgbtq[Title/Abstract]) OR (indigene[Title/Abstract]) OR (seniors[Title/Abstract]) OR (children[Title/Abstract]) OR (youth[Title/Abstract]) OR (neurodiversity[Title/Abstract]) OR (refugees[Title/Abstract]) OR (asylum[Title/Abstract]) OR (spatial disparity[Title/Abstract]) OR (spatial disparities[Title/Abstract]) OR (neighborhood[Title/Abstract]) OR (neighborhoods[Title/Abstract]) OR (neighbourhood[Title/Abstract]) OR (neighbourhoods[Title/Abstract]) OR (racism[Title/Abstract]) OR (ethnic minority[Title/Abstract]) OR (ethnic minorities[Title/Abstract]) OR (sinti[Title/Abstract]) OR (roma[Title/Abstract]) OR (religion[Title/Abstract]) OR (islamophobia[Title/Abstract]) OR (antisemitism[Title/Abstract]) OR (antiziganims[Title/Abstract]) OR (sexism[Title/Abstract]) OR (capitalism[Title/Abstract]) OR (patriarchy[Title/Abstract]) OR (colonialism[Title/Abstract]) OR (group based discrimination[Title/Abstract]) OR (homelessness[Title/Abstract])OR (education[Title/Abstract]) OR (inequality[Title/Abstract])) AND ((systematic review[Title/Abstract]) OR (meta-analysis[Title/Abstract]) OR (meta analysis[Title/Abstract]) OR (literature review[Title/Abstract]) OR (scoping review[Title/Abstract]))

Focus topic: Resilience factors for mental stability in the context of climate change in Germany
((climate change[Title/Abstract]) OR (global warming[Title/Abstract]) OR (climate crisis[Title/Abstract]) OR (climate[Title/Abstract]) OR (greenhouse effect[Title/Abstract])) AND ((resilience[Title/Abstract]) OR (protective factor[Title/Abstract]) OR (adaption[Title/Abstract]) OR (coping[Title/Abstract]) OR (adjustment[Title/Abstract]) OR (risk factor[Title/Abstract])) AND ((mental[Title/Abstract]) OR (psychological[Title/Abstract]) OR (well-being[Title/Abstract]) OR (well being[Title/Abstract]) OR (behavioral[Title/Abstract]) OR (behavioural[Title/Abstract])OR(psychosocial[Title/Abstract]) OR (life satisfaction[Title/Abstract])OR(quality of life[Title/Abstract])) AND ((german[Title/Abstract]) OR (germany[Title/Abstract]) OR (deutsch[Title/Abstract]) OR (Deutschland[Title/Abstract]))
((climate change[Title/Abstract]) OR (global warming[Title/Abstract]) OR (climate crisis[Title/Abstract]) OR (climate[Title/Abstract]) OR (greenhouse effect[Title/Abstract])) AND ((resilience[Title/Abstract]) OR (protective factor[Title/Abstract]) OR (adaption[Title/Abstract]) OR (coping[Title/Abstract]) OR (adjustment[Title/Abstract]) OR (risk factor[Title/Abstract])) AND ((mental[Title/Abstract]) OR (psychological[Title/Abstract]) OR (well-being[Title/Abstract]) OR (well being[Title/Abstract]) OR (behavioral[Title/Abstract]) OR (behavioural[Title/Abstract])OR(psychosocial[Title/Abstract]) OR (life satisfaction[Title/Abstract])OR(quality of life[Title/Abstract])) AND ((systematic review[Title/Abstract]) OR (meta-analysis[Title/Abstract]) OR (meta analysis[Title/Abstract]) OR (literature review[Title/Abstract]) OR (scoping review[Title/Abstract]))
